# *Lactobacillus rhamnosus* GG Intake Modifies Preschool Children’s Intestinal Microbiota, Alleviates Penicillin-Associated Changes, and Reduces Antibiotic Use

**DOI:** 10.1371/journal.pone.0154012

**Published:** 2016-04-25

**Authors:** Katri Korpela, Anne Salonen, Lauri J. Virta, Minna Kumpu, Riina A. Kekkonen, Willem M. de Vos

**Affiliations:** 1 Department of Bacteriology and Immunology, Immunobiology Research Programme, Faculty of Medicine, University of Helsinki, Helsinki, Finland; 2 Research Department, Social Insurance Institution, Turku, Finland; 3 R&D, Valio Limited, Helsinki, Finland; 4 Laboratory of Microbiology, Wageningen University, Wageningen, the Netherlands; The Foundation for Medical Research, INDIA

## Abstract

Antibiotic use is considered among the most severe causes of disturbance to children’s developing intestinal microbiota, and frequently causes adverse gastrointestinal effects ranging from mild and transient diarrhoea to life-threatening infections. Probiotics are commonly advocated to help in preventing antibiotic-associated gastrointestinal symptoms. However, it is currently unknown whether probiotics alleviate the antibiotic-associated changes in children’s microbiota. Furthermore, it is not known how long-term probiotic consumption influences the developing microbiota of children. We analysed the influence of long-term *Lactobacillus rhamnosus* GG intake on preschool children’s antibiotic use, and antibiotic-associated gastrointestinal complaints in a double blind, randomized placebo-controlled trial with 231 children aged 2–7. In addition, we analysed the effect of *L*. *rhanmosus* GG on the intestinal microbiota in a subset of 88 children. The results show that long-term *L*. *rhamnosus* GG supplementation has an influence on the composition of the intestinal microbiota in children, causing an increase in the abundance of *Prevotella*, *Lactococcus*, and *Ruminococcus*, and a decrease in *Escherichia*. The treatment appeared to prevent some of the changes in the microbiota associated with penicillin use, but not those associated with macrolide use. The treatment, however, did reduce the frequency of gastrointestinal complaints after a macrolide course. Finally, the treatment appeared to prevent certain bacterial infections for up to 3 years after the trial, as indicated by reduced antibiotic use.

***Trial Registration***: ClinicalTrials.gov NCT01014676

## Introduction

Antibiotics account for the majority of prescription medication used by children in western countries [1] and relatively frequently cause diarrhoea in this demographic [2], which indicates that antibiotic use influences intestinal health. Hence, antibiotic use has been implicated as the strongest and the most common cause of disturbance to the intestinal microbiota [3]. It has been suggested that due to their impact on the commensal microbiota and thereby reduced colonization resistance, antibiotics predispose the patient to life-threatening intestinal infections with pathogens such as *Clostridium difficile* [4]. Several studies have shown changes in the intestinal microbiota in response to oral antibiotic treatments in adult humans [5–8] and in animals [9–11]: the immediate effect is usually a reduction in total bacterial abundance and diversity, after which antibiotic-resistant species or opportunistic species with fast intrinsic growth rates repopulate the vacated niches, altering the ecological balance in the intestine, the metabolic functions of the microbiota and the host-microbe interactions and host immune function [11, 12]. Bile acid and steroid metabolism has been shown to decrease, and the metabolism of sugars and starch to increase after an antibiotic course [11].

Animal experiments have demonstrated that early-life antibiotic use disrupts the microbiota and consequently immune function and metabolism, predisposing to the development of obesity [13,14] and allergic disease [15,16]. Recent studies have indicated that the growth-promoting and obesogenic effects of antibiotic use also are manifested in humans [17–20]. The association between antibiotic use and the development of asthma in children has been established, although causality is yet to be confirmed [21]. Due to these potentially detrimental changes, there is a need to mitigate the damage antibiotics cause on the symbiotic microbiota. One commonly advocated option is the use of specific microorganisms that are marketed as probiotics.

Probiotics have been recently redefined as live microorganisms that when administered in adequate amounts confer a health benefit on the host [22]. Lactic acid bacteria and bifidobacteria are among the most commonly used probiotics. The evidence for their efficacy varies between strains, aimed health outcomes, and study cohorts [23,24]. *Lactobacillus rhamnosus* GG is one of the best studied probiotics [25] and meta-analyses have shown this bacterium to be effective in the treatment of many gastrointestinal illnesses in children: it reduces gastrointestinal pain [26], *Clostridium difficile* -associated diarrhoea [27], hospital-acquired diarrhoea [28], antibiotic-associated diarrhoea [29], and the duration of infectious diarrhoea [30]. Short-term probiotic use has not been found to result in large changes in faecal microbiota composition of adults [31,32]. This suggests that the mode of action may be related to altered microbial metabolism or direct interaction with the host [24]. *L*. *rhamnosus* GG produces pili decorated with the mucus-binding protein SpaC [33]. The pili have been implicated in various signalling effects, including those with the immune system [24,34]. There is some indication that probiotics may alleviate the antibiotic-induced alterations in the composition of the intestinal microbiota [35,36].

The long-term effects of continuous probiotic use on the developing intestinal microbiota of children, and its potential to protect from antibiotic-associated changes in the microbiota, are not well documented. *L*. *rhamnosus* GG is available in various food products and pharmaceutical formulations, making the continuous consumption of this strain very common. In this study we address the effect of long-term consumption of *L*. *rhamnosus* GG on the health outcome and microbiota of a cohort of preschool children by utilizing antibiotic purchase records and culture-independent microarray analysis of the faecal microbiota.

## Materials And Methods

### Participants and study design

The study is based on a double-blind placebo-controlled trial, conducted during the winter 2009–2010, investigating the effect of *L*. *rhamnosus* GG supplementation on the occurrence of viral respiratory infections in children aged 2–6 years [37]. The study was approved by the Ethics Committee of Joint Authority of Kainuu Region and registered to http://clinicaltrials.gov with the identifier NCT01014676. The parents provided written informed consent. The participants were randomly assigned into probiotic and placebo treatment groups, both receiving three *ad libitum* doses of milk daily, on average 400 ml per day. The probiotic treatment group received milk containing *L*. *rhamnosus* GG (*L*. *rhamnosus* GG; approximately 10^6^ cfu/ml, Valio Gefilus milk 1% fat), and the placebo group received similar milk (Valio 1%) without the probiotic. The participants were guided not to use probiotic products during the intervention and three weeks prior to the intervention. The intervention continued for seven months (October 2009–April 2010). All participants attended a health check and were asked to provide a faecal sample at the beginning and end of the intervention period. Compliance with the milk intake in both groups was monitored by the day care staff and parents, who recorded the daily intake. Moreover, parents kept daily symptom diaries throughout the intervention, marking-down various gastrointestinal and respiratory complaints. For the current study, the frequency of gastrointestinal complaints (pain, bloating, diarrhoea, constipation, flatulence) per day was calculated before, during and after antibiotic treatment for the children who used antibiotics during the intervention to assess the ability of the probiotic to reduce antibiotic-associated gastrointestinal complaints.

Originally a total of 501 children completed the study. Exclusion criteria in the original study were milk allergy, lactose intolerance, congenital heart disease requiring regular medication, malignant diseases, cytostatic treatment, use of biological rheumatic medication, continuous microbial medication, regular use of oral corticosteroids, diabetes and simultaneous participation in other clinical trials. Written informed consent (by the parents) to access the antibiotic purchase records was provided by 231 children. The national individual-based drug purchase registry of the Finnish Social Insurance Institute was utilized to obtain information on antibiotic purchases during the intervention and for three years after the intervention (until the end of 2012). The collection of antibiotic purchase records was approved by the ethical boards of the Helsinki and Uusimaa Hospital Region, and the Social Insurance Institute. We categorized the antibiotics into penicillins, (phenoxymethylpenicillin, amoxicillin, amoxicillin with clavulanic acid), 1^st^ generation cephalosporins, macrolides (azithromycin and clarithromycin), and sulphonamide-trimethoprim. Antibiotics in Finland are only available by prescription by a medical doctor, but prescriptions can be made without a positive bacterial culture. Therefore, the antibiotic purchases reflect bacterial infections, but the correlation may not be perfect. It is possible that viral infection are sometimes incorrectly diagnosed as bacterial and treated with antibiotics.

**Fig 1:**
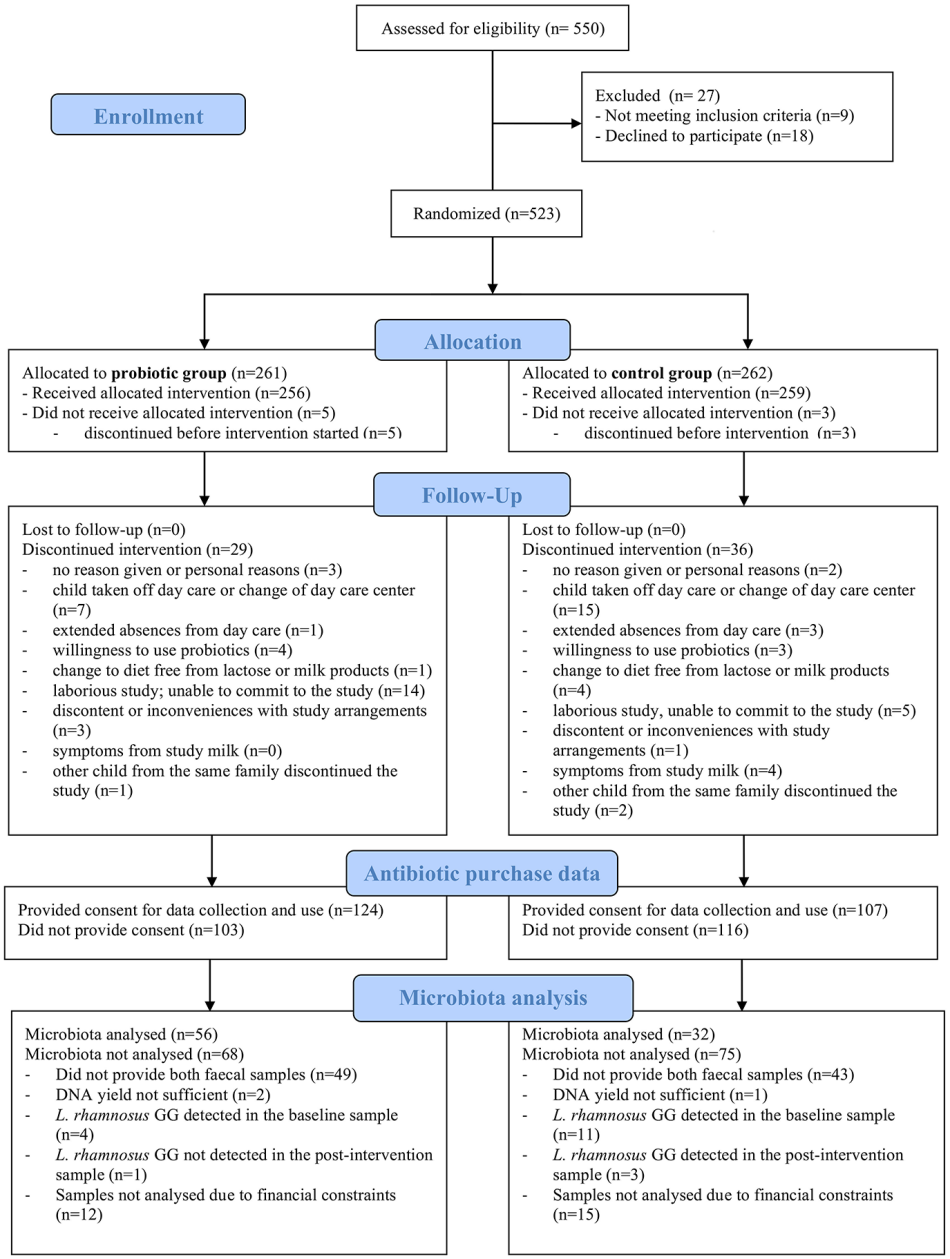
Flow-chart of the study cohort.

For the microbiota analysis, 88 children were selected based on the following criteria: availability of both faecal samples; adequate DNA yield from both faecal samples; and compliance with the probiotic/placebo treatment based on PCR-detection (or absence) of *L*. *rhamnosus* GG in the samples (Fig 1). The PCR-test was conducted on all samples, including the control samples to ensure that had not consumed products containing LGG. In the original study, the children were randomized into the two treatment groups; however, for this study a sub-cohort was selected and therefore we considered the possibility that the children were non-randomly distributed between the groups in this sub-cohort. However, we found no significant differences between the children included in the microbiota analyses and those not included (Table 1). Furthermore, we found no significant baseline differences in age, duration of breastfeeding, weight, height, lifetime antibiotic use, or the number of cases with asthma or allergies between the placebo and LGG group among the children whose microbiota was analysed (Tables 1 and 2). Nevertheless, we used each child’s own baseline microbiota sample as a covariate in the models, to control for any potentially unknown confounders affecting the microbiota composition.

**Table 1:** Characteristics of the study cohort (N = 231). Control = received neither antibiotics nor *L*. *rhamnosus* GG during the intervention; Pen = received penicillin; Mac = received macrolide and/or penicillin; LGG = received *L*. *rhamnosus* GG but no antibiotics; Pen+LGG = received *L*. *rhamnosus* GG and penicillin; Mac+LGG = received *L*. *rhamnosus* GG and macrolide and/or penicillin.

	Control	Pen	Mac	LGG	Pen+LGG	Mac+LGG	Microbiota analysed	Microbiota not analysed
N	18	5	9	34	13	9	88	143
Age (months)	66.3	39.4	48.9	67	57.5	68.9	62.2	52.3
Duration of breastfeeding (months)	8.1	6.9	8	8.5	7.8	8.7	8.2	9.1
Weight (kg)	21.4	14.9	15.7	20.1	17.1	21.9	19.4	18.5
Height (cm)	110.3	92.4	96.5	109.4	102.8	114.1	106.8	105
Lifetime antibiotic courses / year	1.2	2.7	3	1.6	1.5	2.3	1.8	1.8
Cases with asthma	0	0	1	0	1	0	2	7
Cases with allergies	3	1	2	5	1	0	12	10

**Table 2:** Antibiotic use before, during, and after the intervention in the LGG group (N = 124) and placebo group (N = 107).

	All antibiotics			Macrolide		Penicillin		Cephalo-sporins		Sulfonamide-trimethoprim	
	LGG		Placebo	LGG	Placebo	LGG	Placebo	LGG	Placebo	LGG	Placebo
Number of children receiving at least one course											
Before		120	102	80	68	112	99	51	50	51	57
During		49	48	14	20	39	35	6	8	6*	**15**
1yr after		72	69	19	26	57	52	20	21	13*	**23**
2yr after		84	84	26*	**37**	65	65	32	28	18	27
3yr after		90	86	30	38	70	65	37	31	20	29
Average number of courses per child											
Before		7.4	7.5	1.6	2.0	4.0	3.4	0.8	0.9	1.0	1.1
During		0.7	1.0	0.1	0.3	0.5	0.5	0.1	0.1	0.1*	**0.2**
1yr after		1.6	2.1	0.2*	**0.4**	1.0	1.0	0.2	0.3	0.2	0.4
2yr after		2.5	3.2	0.3*	**0.7**	1.4	1.5	0.4	0.5	0.3	0.6
3yr after		2.8	3.5	0.4*	**0.8**	1.6	1.5	0.5	0.6	0.4	0.6

Significant differences between the LGG and the placebo groups are indicated by * and bold font. The significance is based on χ^2^-test (antibiotic use frequencies) and t-test (average number of courses).

For the microbiota analysis, the 88 children were categorized into 6 treatment groups based on their antibiotic purchases during the 7-month intervention (Table 1): Control (no antibiotic or probiotic treatments), Pen (penicillin use during the intervention), Mac (macrolide use during the intervention, some also used penicillins), LGG (*L*. *rhamnosus* GG group), Pen+LGG (*L*. *rhamnosus* GG + penicillin), and Mac+LGG (*L*. *rhamnosus* GG + macrolide). The use of sulfonamide-trimethoprim (N = 7) and cephalosporins (N = 4) were so uncommon during the intervention that their association with the microbiota could not be assessed.

### Processing of the faecal samples

The faecal samples were collected at home and transported immediately to the study centre for storage in -70°C, or were initially stored in the home freezer for 0.5–78 hours (on average 19 hours) before being transported to the study center. DNA was extracted from the faecal samples with the Promega Genomic Wizard DNA Purification Kit (Promega, Madison, WI, USA), using a modified protocol developed for bacterial DNA extraction from faecal samples [38]. Compliance with the probiotic/placebo treatment was confirmed by testing the post-intervention samples with a qPCR specific for the *L*. *rhamnosus* GG strain [38]. Only compliant cases were included in microbiota analysis. The bacterial composition of the participants’ microbiota was studied using a phylogenetic microarray, the Human Intestinal Track Chip (HITChip), which is specifically designed for the analysis of the human intestinal microbiota [39]. The microarray consists of oligonucleotide probes targeting the hyper-variable regions V1 and V6 of the 16S rRNA gene, allowing the identification and relative quantification of not only previously cultured and named, but also uncultured bacterial phylotypes. Microarray analysis of the bacterial DNA was performed essentially as described previously [39]. The signal intensities of the oligonucleotide probes were translated into abundances of 1038 species-level phylotypes, 130 genus-like taxa, and 23 higher level taxa (9 phyla; Firmicutes divided down to Clostridium clusters and Bacilli) using the fRPA pre-processing algorithm [40]. The genus-like taxonomy is formed by grouping together related (>90% similarity of 16S rRNA gene sequence) organisms, which is the established cut-off for 16S-based genus annotation, and which has been shown to correlate well with FISH (fluorescence *in situ* hybridization) -based quantitation of bacterial genera [39]. The groups are named according to the nearest cultured relative. The microbiota data were transformed into relative abundances by dividing the signal intensities of each taxon by the total intensity of the sample. The microarray data are available in S2 Table.

### Statistical analyses

To investigate the associations between *L*. *rhamnosus* GG treatment and antibiotic purchases (as a proxy for bacterial infections) from the beginning of the intervention in 2009 until the end of 2012 we included 231 children (placebo group, N = 107; *L*. *rhamnosus* GG group, N = 124). Survival analysis (with survival regression models, assuming the exponential distribution) was conducted to test if the treatment groups differed in their persistence without antibiotics. The end outcome in the models was the first antibiotic purchase. Negative binomial models were used to analyse the cumulative number of courses purchased by different time points.

The number of gastrointestinal (GI) complaints before, during and after antibiotic courses was analysed in a sub-cohort including 96 cases that had purchased antibiotics during the intervention (placebo group, N = 47; *L*. *rhamnosus* GG group N = 49). Using a paired-sample Wilcoxon-test, we tested whether the frequency of GI complaints (average number of complaints/day) was higher after an antibiotic course than before in the groups Pen, Mac, Pen+LGG, and Mac+LGG. Using the Kruskal-Wallis test, we tested whether the difference in the GI complaint frequency before and after an antibiotic course was different between the Pen and Pen+LGG groups, and between the Mac and Mac+LGG groups.

Species diversities were calculated for each faecal sample as the inverse Simpson index of the species-level data. Microbiota stability was calculated as the Pearson correlation between the pre- and post-intervention sample. The principal coordinates analysis was conducted with the log-transformed species-level data, using Pearson correlation distances. The influence of the probiotic and antibiotic treatments on the genus-level microbiota was analysed with negative binomial models, controlling for the baseline microbiota composition, and thereby any factor that may have influenced the baseline composition such as age, health, and antibiotic use history, by including the baseline abundance of the focal bacterial group in the model. Thus further stratification was not necessary. The timing of the antibiotic course during the intervention had no impact on the estimated effect of the antibiotic, and was therefore excluded from the analysis. Due to the large number of genus-level bacterial groups (130), only the false discovery rate—corrected p-values <0.05 were considered as significant and were reported. The genus-level bacterial groups significantly associated with the treatments were clustered based on their response profiles (estimated effects of the treatments from the negative binomial models) to visualize, which bacterial groups respond to the treatments similarly.

All statistical analyses were conducted with the program R [41], using the packages vegan [42], MASS [43], nlme [44], and survival [45].

## Results

### *L*. *rhamnosus* GG supplementation caused a reduction in antibiotic use

Using the Finnish drug purchase registry, we were able to accurately follow the antibiotic use of the study children during and for nearly 3 years after the intervention. The treatment groups did not differ in prior antibiotic use or prevalence of asthma or allergies (Tables 1 and 2).

**Fig 2:**
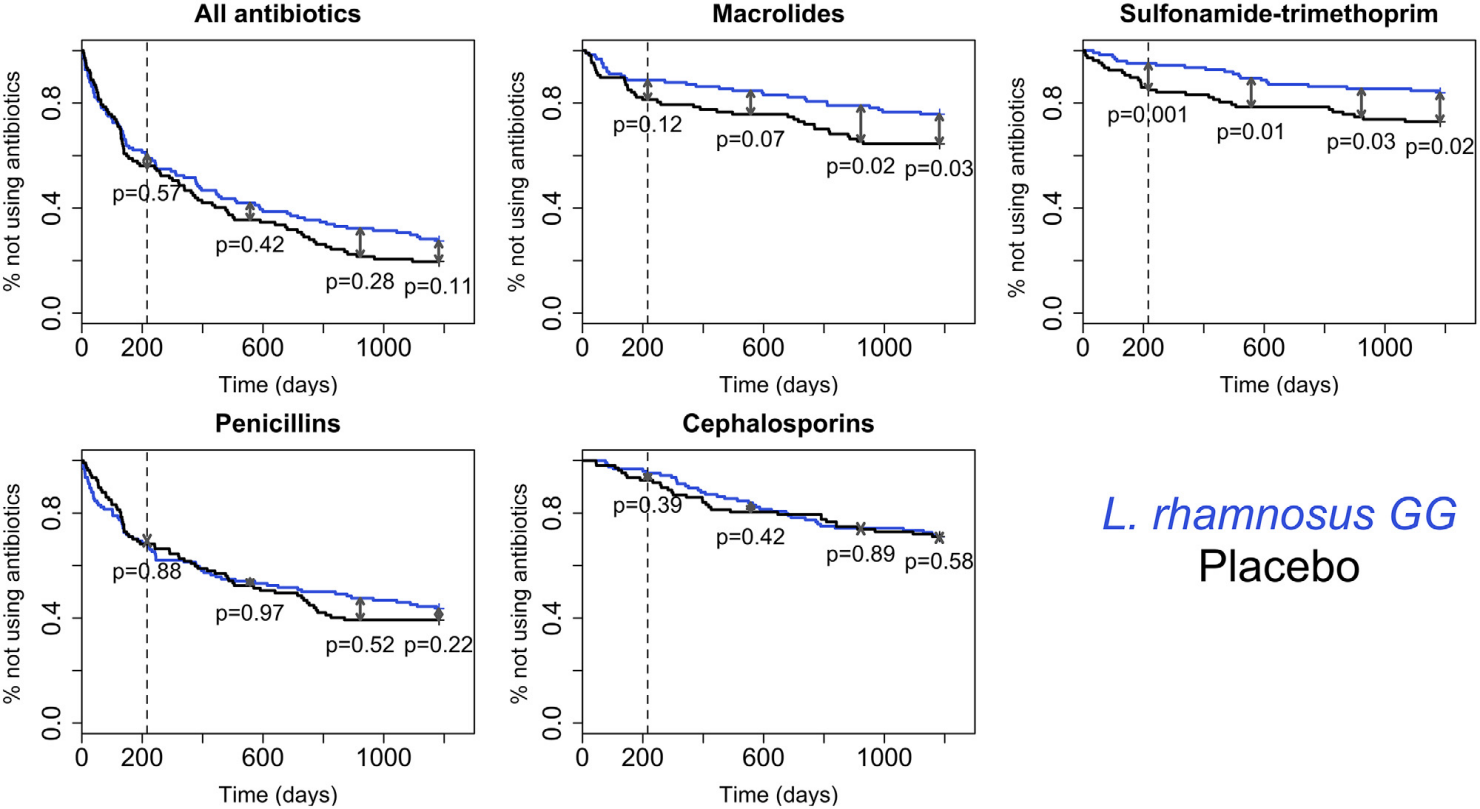
Incidence of antibiotic use (%) in the placebo and *L*. *rhamnosus* GG groups. The dashed line shows the end of the intervention. The p-values indicate the significance of the difference (indicated by the arrows at different time points, based on survival regression models) in antibiotic use between the groups at the end of the intervention, 1 year after the intervention, 2 years after the intervention, and 2.7 years after the intervention.

The prevalence of total antibiotic use during the intervention did not significantly differ between the treatment groups (Fig 2, Table 2). During the intervention, 44% of the placebo group and 40% of the *L*. *rhamnosus* GG group received antibiotics. However, the prevalence of sulfonamide-trimethoprim use was significantly reduced in the *L*. *rhamnosus* GG group (relative risk, RR = 0.34, 95% confidence interval 0.14–0.85, Fig 2, Table 2). During the follow-up period after the intervention, the difference in antibiotic use between the groups gradually increased (Fig 2). There was a significant difference in the proportion of children treated with macrolides (RR = 0.68, 95% CI = 0.46–1.02) and sulfonamide-trimethoprim (RR = 0.6, 95% CI = 0.36–0.99) by the end of the follow-up period (Fig 2). The difference in penicillin and cephalosporin use was smaller and not significant.

**Fig 3:**
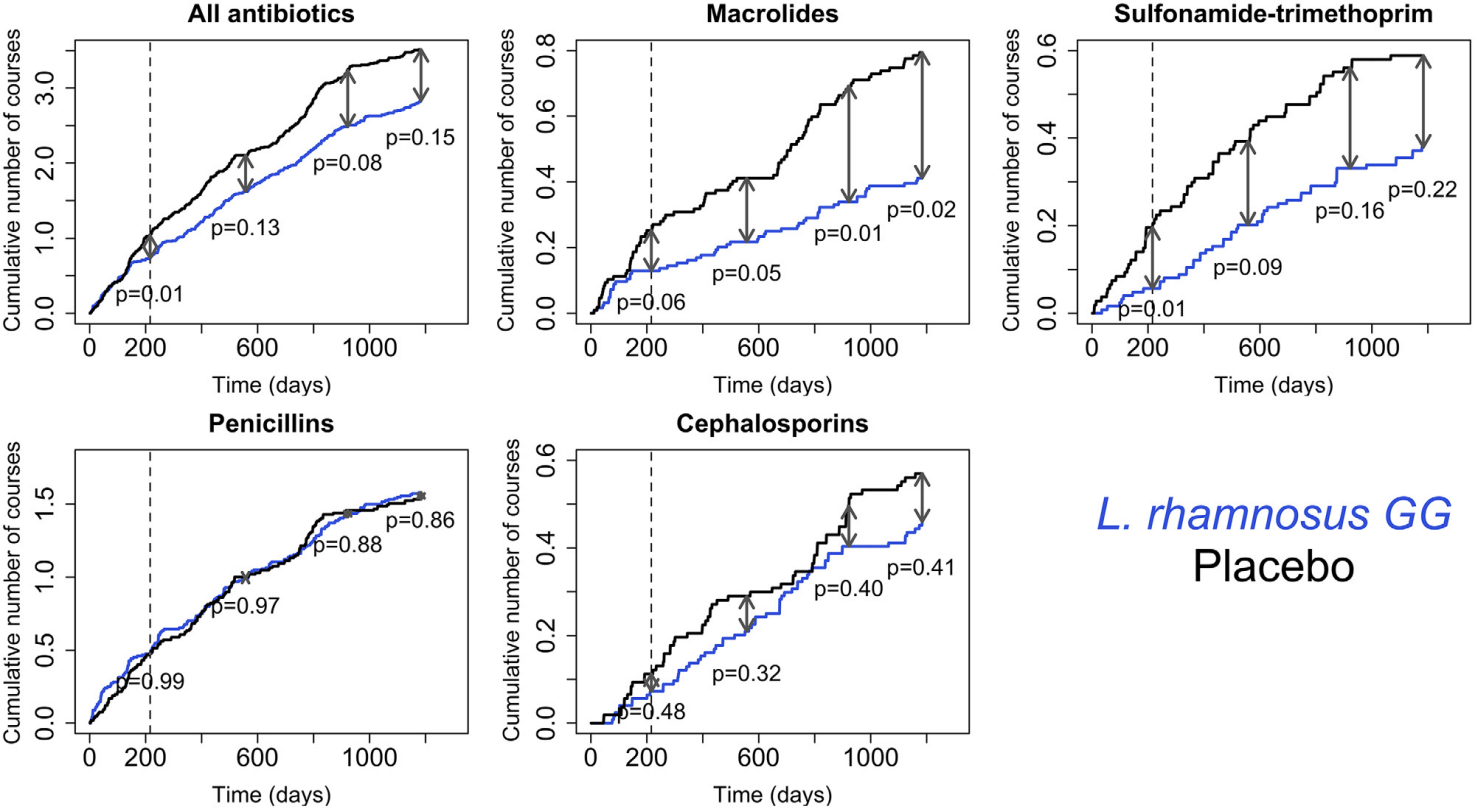
Cumulative number of antibiotic courses per person in the placebo and *L*. *rhamnosus* GG groups. The dashed line shows the end of the intervention. The p-values indicate the significance of the difference (indicated by the arrows at different time points, based on negative binomial models) in antibiotic use between the groups at the end of the intervention, 1 year after the intervention, 2 years after the intervention, and 2.7 years after the intervention.

The number of antibiotic courses purchased per child followed similar patterns (Fig 3): the cumulative number of antibiotic courses was consistently lower in the *L*. *rhamnosus* GG group, and the difference was especially clear in macrolide and sulfonamide-trimethoprim courses. The *L*. *rhamnosus* GG group received 49% fewer macrolide and 72% fewer sulfonamide-trimethoprim courses per person during the intervention. By the end of the follow-up, the *L*. *rhamnosus* GG group had received 48% fewer macrolide and 36% fewer sulfonamide-trimethoprim courses per person.

### *L*. *rhamnosus* GG protected against macrolide-associated gastrointestinal disturbance

**Fig 4:**
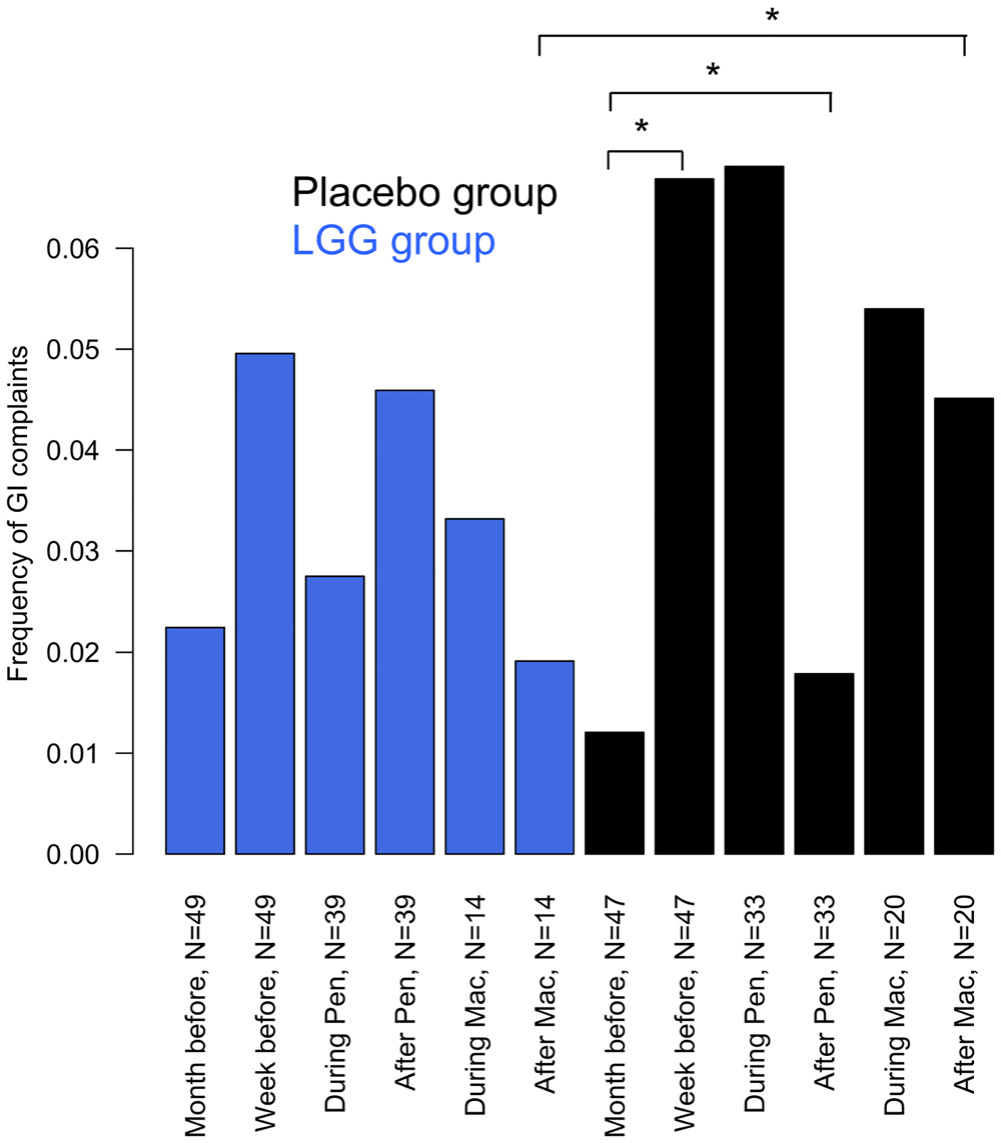
Frequency of gastrointestinal complaints per day per person during the intervention. The frequency of complaints was calculated separately for the following periods: during the month before any antibiotic course, during the week before any antibiotic course, during a penicillin or a macrolide course, and during the month after a penicillin or a macrolide course. Some children received several courses and therefore contributed to the calculation of penicillin and macrolide-associated symptoms. Significant differences based on non-parametric Kruskal-Wallis (when comparing groups) and paired-sample Wilcoxon (when comparing different time periods in the same individuals) -tests are shown.

There frequency of gastrointestinal complaints in the children that received antibiotics during the intervention (number of complaints per day per child) was on average highest during the week before the start of an antibiotic course (Fig 4), with no differences between the *L*. *rhamnosus* GG and placebo group (p = 0.96). There was a borderline significant increase in the frequency of complaints during the week before a course compared to the previous month in the placebo group (p = 0.04) and in the *L*. *rhamnosus* GG group (p = 0.11). Neither penicillin nor macrolide courses were associated with an immediate increase in the frequency of symptoms during the course in either treatment group. However, during the month after a macrolide course, there was a significantly higher frequency of complaints in the placebo group as compared to the probiotic group (p = 0.03).

### *L*. *rhamnosus* GG intake modified the children’s faecal microbiota

**Fig 5:**
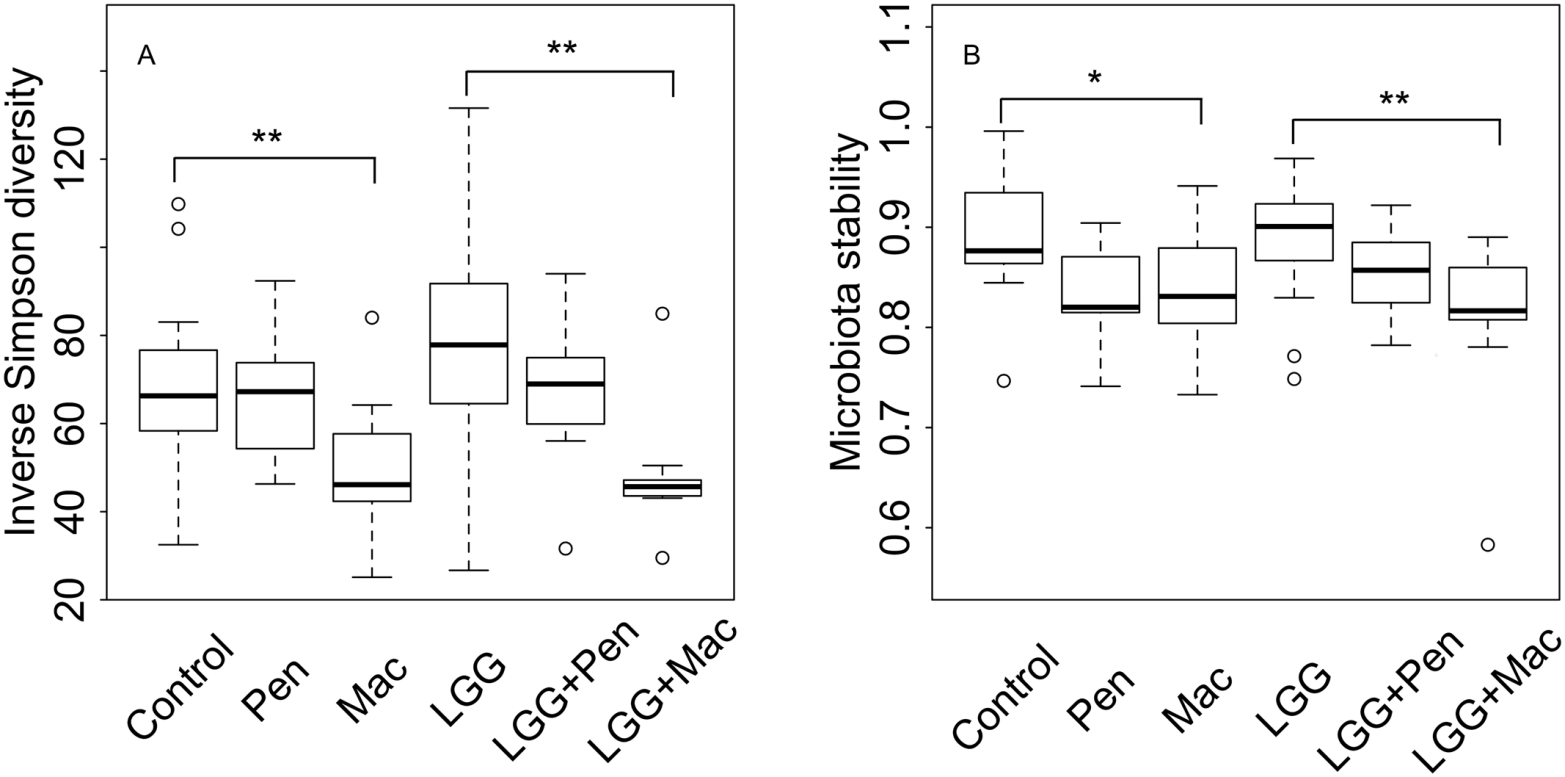
Microbiota diversity and stability by treatment group. Diversity after the intervention was calculated as the Inverse Simpson index (panel A), and microbiota stability as the Pearson correlation between the pre- and post-intervention sample (panel B). Significant differences based on analysis of variance are shown.

In the children that were not treated with antibiotics, the *L*. *rhamnosus* GG treatment caused microbial changes (S1 Table), although the diversity and stability were unaffected (Fig 5). According to permutational multiavariate analysis of variance, the treatment explained 4% of the inter-individual variation in the species-level microbiota composition (p = 0.001) among the non-antibiotic-treated children. The abundance of relatives of *Lactococcus*, *Lactobacillus gasseri*, *Ruminococcus lactaris*, uncultured Mollicutes, *Prevotella melaninogenica* and *P*. *oralis* were significantly elevated in the probiotic group, while the abundance of relatives of *Eubacterium cylindroides*, *Clostridium ramosum*, and *Escherichia coli* were reduced (S1 Table).

### *L*. *rhamnosus* GG did not protect from macrolide-associated changes in the microbiota

**Fig 6:**
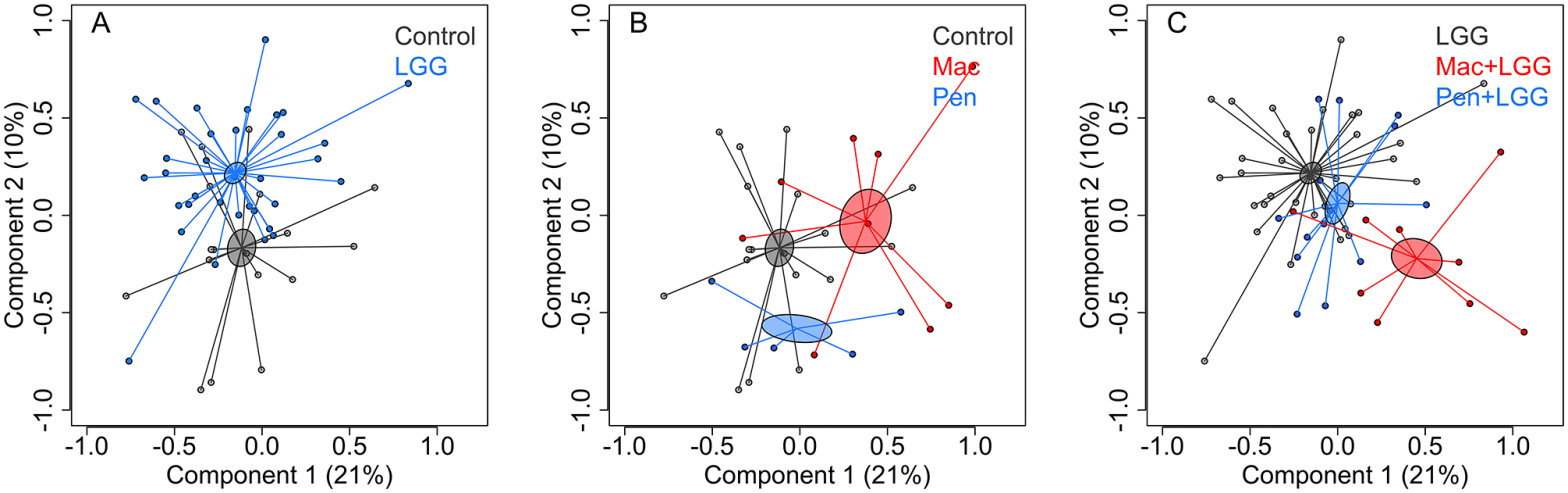
Microbiota composition by treatment group. Composition in the placebo group (panel A) and *L*. *rhamnosus* GG group (panel B). The component scores were calculated using principal coordinates analysis of the Pearson correlation distances in the species-level data. All samples were included in the same analysis, and the groups are shown in different panels for clarity.

Antibiotic use was strongly associated with the post-intervention microbiota composition, explaining 14% of the inter-individual variation in both the placebo and probiotic groups (permutational multivariate analysis of variance, p = 0.001). In both groups, the microbiota of the macrolide-treated children deviated most strongly from the non-antibiotic treated children. Macrolide use was associated with reduced diversity and stability of the microbiota (Fig 5). The effect of penicillins on the microbiota was limited and the microbiota of the penicillin users resembled the microbiota of the non-antibiotic-users (S1 Table, Figs 5 and 6).

**Fig 7:**
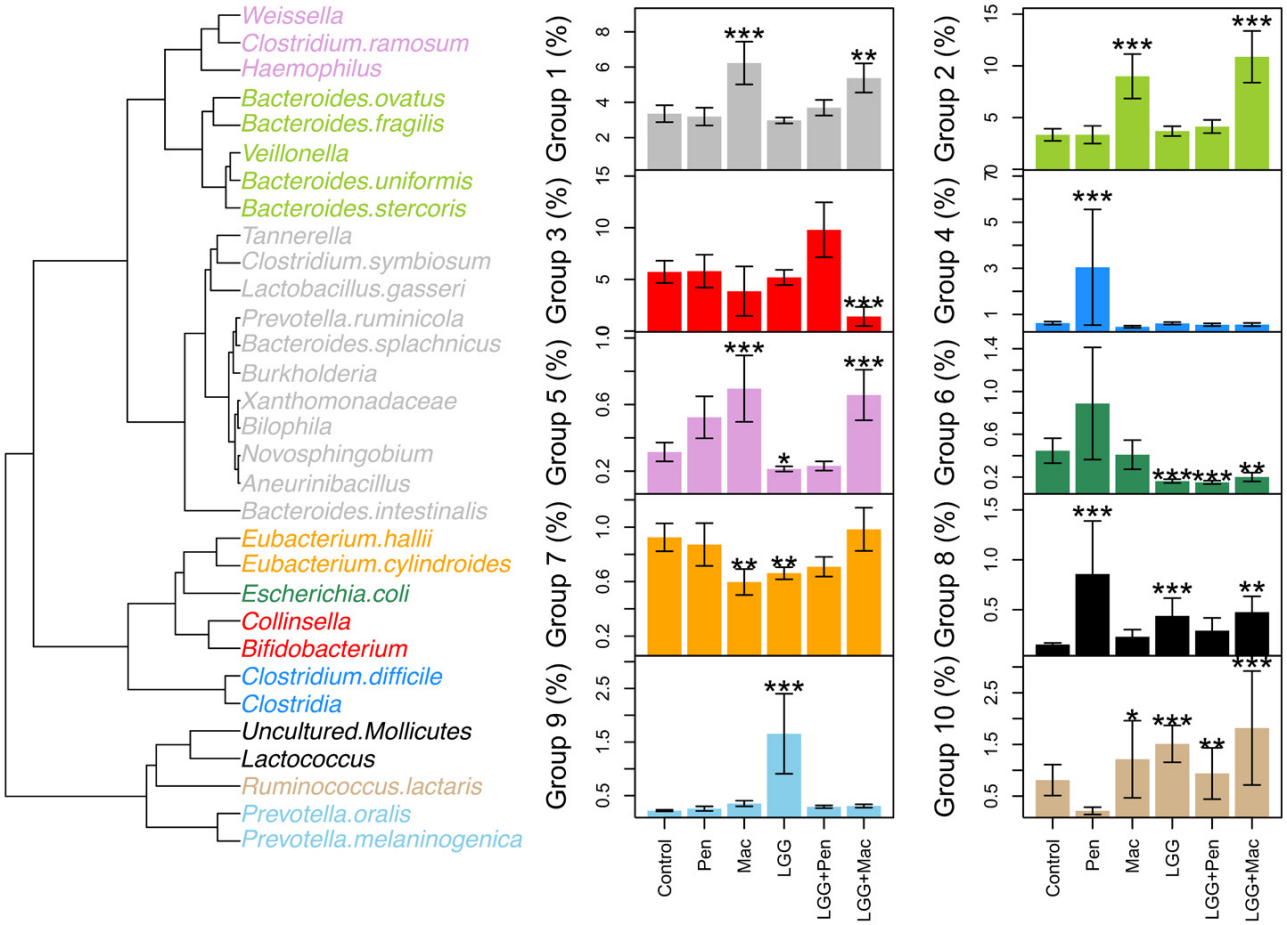
Bacterial groups associated with the treatments. Genus-level bacterial groups significantly associated with at least one of the treatments (*L*. *rhamnosus* GG or antibiotics), clustered based on their response profile to the *L*. *rhamnosus* GG and antibiotics. The average total abundances (± standard error) of the clusters in the different treatment groups are shown in the barplots. Significance of the difference from the control group (based on negative binomial models) are indicated by the asterisks: * p<0.05; ** p<0.01; *** p<0.001.

Most of the macrolide users, regardless of probiotic use, had a distinct microbiota composition with low diversity (Fig 5), and a high abundance of *Bacteroides* spp. (Fig 7). To see which bacterial groups responded similarly to the treatments, the bacterial groups significantly associated with either antibiotic or *L*. *rhamnosus* GG treatment were clustered into 10 groups based on their response profiles (Fig 7). A diverse group of taxa belonging to Bacteroidetes, Bacilli, and Proteobacteria clustered together (Fig 7, grey). Their total abundance was positively associated with macrolide use. Similarly, the abundance of several *Bacteroides* groups, together with *Veillonella* (Fig 7, light green), as well as the abundance of *Haemophilus*, *Clostridium ramosum*, and *Weissella* (Fig 7, violet) was positively associated with macrolide use. Conversely, the abundance of the Actinobacterial genera *Bifidobacterium* and *Collinsella*, was negatively associated with macrolide use, especially in the *L*. *rhamnosus* GG group (Fig 7, red). However, the *L*. *rhamnosus* GG treatment appeared to prevent the penicillin-associated increase of Clostridium cluster I (the genus-level group *Clostridium* sensu stricto), and the relatives of *Clostridium difficile*, uncultured Mollicutes, and *Haemophilus* spp. (S1 Table, Fig 6).

## Discussion

The influence of long-term *L*. *rhamnosus* GG supplementation on antibiotic use, antibiotic-associated gastrointestinal complaints, and on the intestinal microbiota was investigated in 2–7 year old children. Contrary to earlier observations in short interventions with adults [31,32], the consumption of *L*. *rhamnosus* GG changed the microbiota composition of the children. Macrolide use was associated with strong changes in the microbiota, which the *L*. *rhamnosus* GG treatment did not prevent completely. However, the intake of *L*. *rhamnosus* GG did appear to prevent some of the penicillin-associated changes in the microbiota. Most intriguingly, the use macrolide and sulphonamide-trimethoprim antibiotics was significantly reduced in the intervention group for up to three years post-intervention.

Several genus-level bacterial groups were significantly associated with the treatment, indicating that the regular consumption of *L*. *rhamnosus* GG for 7 months has the potential to alter certain aspects of the intestinal microbiota in children. The observed changes occurred largely (but not exclusively) among the bacteria residing in the small intestine: species related to *Lactococcus* and *Lactobacillus gasseri* increased in abundance by 4.5-fold (p<0.0001) and 1.9-fold (p = 0.005), respectively, while the relatives of *Escherichia coli* decreased by 2.5-fold (p<0.0001). The daily consumption of *L*. *rhamnosus* GG is likely to increase the concentration of its main metabolic product, lactic acid, in the small intestine, which may promote other lactic acid bacteria and conversely reduce the abundance of competing Proteobacteria that are less acid-tolerant. This may be a beneficial change, as many species and strains related to *E*. *coli* are potentially pathogenic and produce the inflammatory lipopolysaccharide (LPS) [46]. However, such changes have not been observed in previous studies with adults [31,32], indicating that the adult microbiota may be more resistant to modulation than the developing microbiota of preschool children, or that long-term supplementation is necessary for changes in the microbiota to occur.

Although the *L*. *rhamnosus* GG intervention appeared to have some beneficial effects on the microbiota and was associated with reduced frequency of GI complaints after a macrolide course, it failed to prevent most of the macrolide-associated changes in the microbiota. This indicates that the protective effect of *L*. *rhamnosus* GG during antibiotic treatment is not caused by stabilization or a faster recovery of the overall microbiota. Macrolide use in both the *L*. *rhamnosus* GG and the placebo group was associated with a clear and consistent pattern of low abundance of the Actinobacterial genera *Bifidobacterium* and *Collinsella*, and a high abundance of *Bacteroides* species related to *B*. *fragilis*, *B*. *ovatus*, *B*. *stercoris*, and *B*. *uniformis* and many groups of Proteobacteria. The only clear benefit of the *L*. *rhamnosus* GG intervention was observed on the relatives of *Eubacterium hallii*, a butyrate-producer [47], which declined in the placebo-treated macrolide users but not in the *L*. *rhamnosus* GG—treated ones. Whether the protective effect of *L*. *rhamnosus* GG on *E*. *hallii* or other butyrate producers during macrolide treatment is general and results in a reduction of gastrointestinal disturbance remains to be verified.

Nearly all children using macrolides had increased levels of *Bacteroides* spp. Many *Bacteroides* species are often macrolide-resistant [48], which may explain their increase. An elevated abundance of *Bacteroides* spp. in children has been associated with increased serum IgE levels [49] and with type I diabetes [50,51]. Species related to *B*. *fragilis* and *B*. *ovatus*, the two groups that were strongly increased in the macrolide-users, have been associated with childhood overweight [52], aberrant immune function, including diabetes [53,54] and intestinal symptoms [55,56].

Pencillin use was associated with a weaker shift in the microbiota composition than macrolide use. The abundance of Clostridium cluster I, relatives of *Clostridium difficile*, uncultured Mollicutes, and *Haemophilus* spp. were all significantly increased in the placebo-treated penicillin-users. However, these changes did not occur in the probiotic group, indicating that the probiotic may be effective in protecting the microbiota against penicillin-associated disturbance and increase in potential pathogens.

Previously, *Lactobacillus-Bifidobacterium* supplementation in adults during *H*. *pylori* eradication therapy (amoxicillin-clarithromycin-lansopratzole) and during amoxicillin treatment has been shown to enable a quicker recovery of the microbiota [35]. Our results show that long-term *L*. *rhamnosus* GG administration may have similar effects in children, enabling a faster recovery from or increased resilience against penicillin-associated disturbance. However, the treatment was ineffective against macrolide-associated disturbance, with *Bifidobacterium* even more strongly depressed by the macrolide in the *L*. *rhamnosus* GG group than in the placebo group. These results suggest that the combination of *Lactobacillus* and *Bifidobacterium* strains may be more effective in preventing antibiotic-associated changes in the microbiota than *Lactobacillus* alone.

Perhaps the most important finding was that the use of macrolide and sulphonamide-trimethoprim antibiotics was significantly and persistently reduced after the intervention for up to 3 years. However, beta-lactam (penicillins and cephalosporins) use was unaffected by the treatment. This suggests that either a type of infection that is treated with macrolide and sulphonamide-trimethoprim, but not with beta-lactam antibiotics, was prevented by the *L*. *rhamnosus* GG treatment, or that beta-lactam purchases are not a good proxy for infections. The latter would be the case if beta-lactam antibiotics were prescribed more leniently than the other antibiotic types, even when a bacterial infection has not been verified. The reduction of antibiotic use is an encouraging result, in terms of the threat of antibiotic resistance and in terms of the impact antibiotic exert on the microbiota. *L*. *rhamnosus* GG intake may prevent antibiotic-associated changes in the microbiota and the potential metabolic and immunological consequences indirectly by preventing infections and thus reducing antibiotic use.

The results confirm that long-term *L*. *rhamnosus* GG supplementation has no clear adverse effects on the microbiota or overall health of 2–7 year old children. It appears to have a beneficial influence on the microbiota composition, to prevent penicillin-associated changes in the microbiota, and to confer a long-term protection against certain infections.

## Supporting Information

S1 Table Effects of probiotic and antibiotic treatments on the post-intervention microbiota. The Fold changes and p-values refer to comparison between the treatment groups and the control group which did not receive any treatments. The bold values indicate a significant (FDR-corrected p-value < 0.05) difference from the control group.
(XLSX)

S2 Table The genus-level microbiota composition in the baseline and post-intervention samples. (XLSX)
